# Family physician practice visits arising from the Alberta Physician Achievement Review

**DOI:** 10.1186/1472-6920-13-121

**Published:** 2013-09-09

**Authors:** Ray Lewkonia, Nigel Flook, Michel Donoff, Jocelyn Lockyer

**Affiliations:** 1 Faculty of Medicine, University of Calgary, Calgary, Alberta, Canada; 2 Faculty of Medicine, University of Alberta, Edmonton, Alberta, Canada

**Keywords:** Clinical performance, Quality improvement, Multi-source feedback

## Abstract

**Background:**

Licensed physicians in Alberta are required to participate in the Physician Achievement Review (PAR) program every 5 years, comprising multi-source feedback questionnaires with confidential feedback, and practice visits for a minority of physicians. We wished to identify and classify issues requiring change or improvement from the family practice visits, and the responses to advice.

**Methods:**

Retrospective analysis of narrative practice visit reports data using a mixed methods design to study records of visits to 51 family physicians and general practitioners who participated in PAR during the period 2010 to 2011, and whose ratings in one or more major assessment domains were significantly lower than their peer group.

**Results:**

Reports from visits to the practices of family physicians and general practitioners confirmed opportunities for change and improvement, with two main groupings – practice environment and physician performance. For 40/51 physicians (78%) suggested actions were discussed with physicians and changes were confirmed. Areas of particular concern included problems arising from practice isolation and diagnostic conclusions being reached with incomplete clinical evidence.

**Conclusion:**

This study provides additional evidence for the construct validity of a regulatory authority educational program in which multi-source performance feedback identifies areas for practice quality improvement, and change is encouraged by supplementary contact for selected physicians.

## Background

In recent years periodic review and assessment of the performance of physicians has become imperative for social accountability and regulatory purposes. This can be done by revalidation or recertification, but this approach may overlook the opportunity to educate and to enhance practice. The Physician Achievement Review (PAR) was introduced in 1999 [1] by the College of Physicians and Surgeons of Alberta (CPSA), which is the provincial medical regulatory authority in Alberta, Canada. PAR has two professional objectives - to promote quality improvement in the practices of individual physicians, and to identify general areas for medical practice improvement in Alberta. The program provides confidential 360-degree multi-source feedback (MSF) referenced to peer groups and derived from questionnaires that are completed by 25 patients, 8 medical colleagues, 8 health co-workers, and a self-assessment. The CPSA has developed the PAR program as an educational quality improvement process which is not administratively linked to the licensing and disciplinary functions of the CPSA.

All physicians who have clinical or procedural contact with patients, images, or tissues are required to participate in PAR every 5 years. Questionnaires have been developed for family physicians and general practitioners, and also for 6 medical specialty groupings [2]. Several studies have been undertaken to assess evidence for the validity and reliability of the instruments [3-6] and this evidence helps to assure physicians regarding the value of performance feedback they receive from the PAR program.

**Figure 1:**
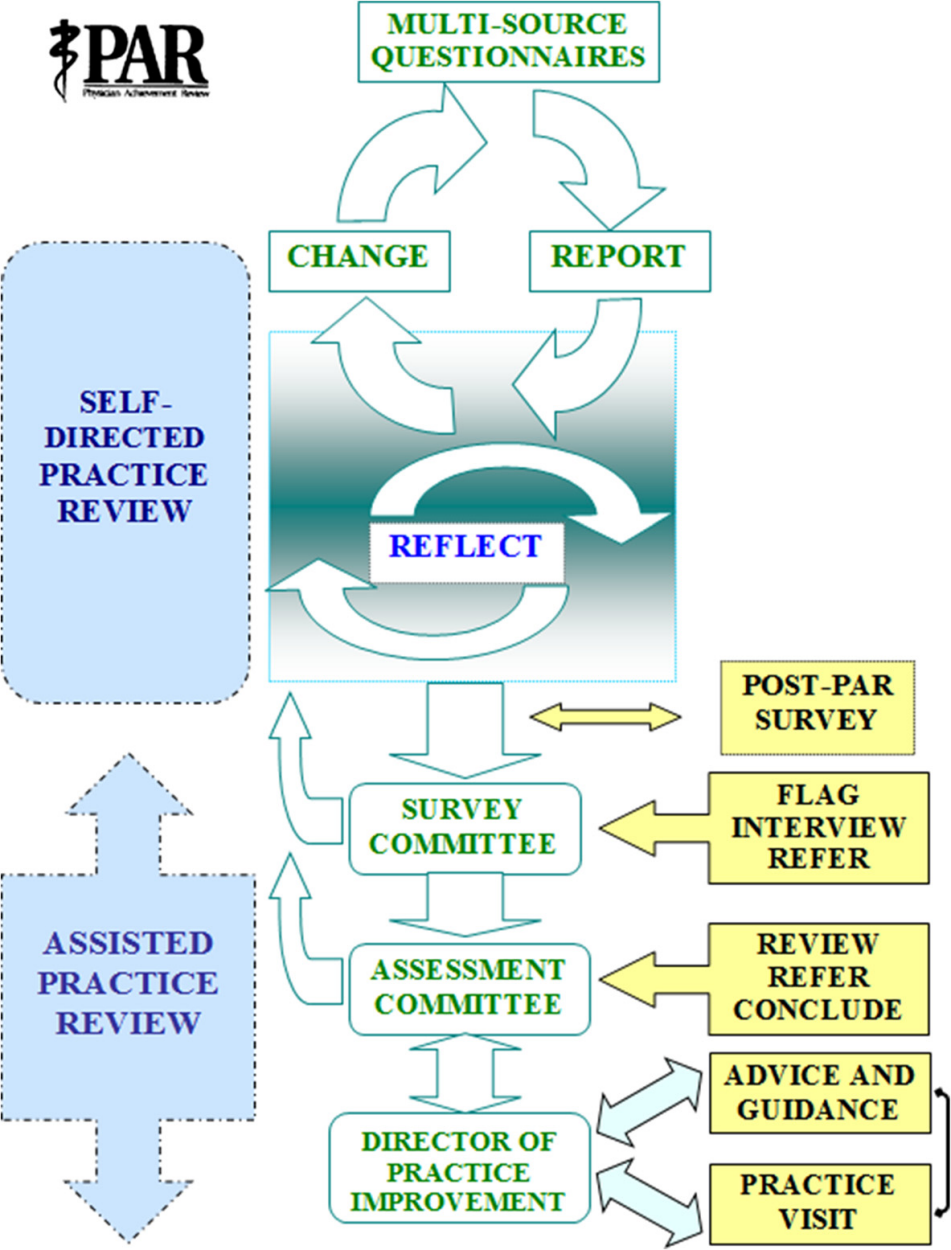
**PAR flow diagram.** The central educational process is reflection on multi-source feedback. Self-directed review is expected to be of value for most physicians. Approximately 20% of physicians are flagged from their PAR data and interviewed by a Survey Committee member, and 2%-4% of physicians are referred to the Assessment Committee and DPI for assisted practice review.

The PAR program has developed an administrative sequence (Figure 1) for physicians who are “flagged” if their questionnaire summary ratings are below 4.0 on 5-point Likert scales or below the 10^th^ centile on peer-referenced scales [2] and this occurs for approximately 20% of family physicians. Flagged physicians are contacted by a member of the PAR Survey Sub-Committee (SSC), who conducts a structured telephone interview to clarify the personal and practice circumstances of the physician, and to discuss the physician’s reactions to their PAR survey report. This dialogue may, for example, disclose valid reasons for physicians being unable to provide the requisite numbers of survey respondents or insight into the reasons for lower ratings, and that self-directed steps in practice improvement are being implemented. Issues causing concern include excessive workload, social problems, and resentment or disbelief of PAR ratings. Based on the SSC member’s report to the committee, a judgment is made regarding the appropriateness of further assessment. Two to four percent of physicians are then referred to the Assessment Sub-Committee (ASC) and are contacted by the Director of Practice Improvement (DPI) for further discussion. This contact is usually followed by a practice visit.

Visits are conducted by trained physicians using several assessment instruments [2] comprising a pre-visit questionnaire that describes the practice environment, patient demographics and practice resources; general office inspection; chart audit of 20 randomly selected medical records; chart-stimulated recall [7] for interactive discussion regarding consultations and management of common clinical presentations, and a continuous patient care questionnaire.

PAR practice visits place emphasis on medical records. Inspection of records has been used for several years as an indicator or proxy for performance [7,8], and additional value has been demonstrated in family practice for chart-stimulated recall conversations in conjunction with chart reviews [9].

Practice visits for family physicians are conducted by one physician who has been trained as a process expert (visits for specialist physicians are conducted by a process expert and a specialty content expert). To the extent possible visitors are matched to the type of practice of the visited physician, so that the visitor observes the practice as a peer. Visitors are not given information from the physician’s PAR questionnaire ratings; they record their findings, but do not provide directive advice. A visitor can describe what is done in their own practice for safety, efficiency and quality. Visit reports are collated and standardised by the Director of Practice Improvement prior to discussion by the Assessment Committee (ASC), which has a membership of physicians with broad clinical and administrative experience and a lay public member. A summary of PAR MSF results is included in the ASC discussion without identification of physicians by name or location. This two-step process balances the implicit judgment of the visitor with the explicit context [10] of the PAR program. A final report and recommendations for practice quality improvement is then produced by the DPI in collaboration with the visited physician. Subsequently the DPI maintains contact with the physician for periods that may vary from a few weeks to several months to assist, guide and confirm recommendations for practice changes*,* and finally the physician is asked to reflect on the value of the PAR process. Confirmation of change is derived from telephone conversations, submitted copies of patient records (with identifiers removed), sometimes with further practice visits, and progress reports to the ASC.

During initial development of practice visit protocols 19 family physicians who had participated in the PAR survey agreed to be visited by two practice visitors, and their paired chart audit results showed close correlation [11]. In addition ten family physicians who received PAR survey commendation ratings above the 90^th^ centiles were visited. The group of commended physicians generally demonstrated excellence in medical record keeping, practice problem-solving skills, and in having special interests in their practice.

The purpose of the current mixed methods study was to analyse final practice visit reports for primary care family practitioners, to explore practice issues that were identified and the recommended changes. The study addressed the following questions: (1) how did the MSF scores received by ‘flagged’ physicians differ from those achieved by a group of unselected family physicians? (2) what themes and subthemes emerged from a qualitative analysis of the practice visit reports? (3) what were the frequencies of the themes and subthemes identified for the group of flagged physicians?

## Methods

Final practice visit reports for certified family physicians and (non-certified) general practitioners whose PAR questionnaire summary data were supplied by the PAR program following removal of all identification except for the physician’s unique PAR number. This retrospective report is based on practice visits done in 2010 and 2011, when the visit protocols, data collected, and electronic formatting were consistent to enable importation into NVIVO8 qualitative analysis software (QSR International).

The reports were analyzed qualitatively [12-14]. The report for each physician's practice visit was entered into the software and a sequential coding approach was followed. Starting with 3 reports, members of the group (RL, JL, HF, NF and MD) independently using open coding and line-by-line analysis [13,14]. The coding scheme was then applied to another five reports using the constant comparative method [13,14] where codes generated in one report, can be applied to previous reports as well as the next report(s) to ensure that new ideas are captured in the coding scheme. Iterative coding of practice visits continued with periodic review (by HF and RL) to modify and clarify the coding structure. Coding continued until no new themes emerged and data saturation was evident. It was then possible for the group to discuss, develop and agree on core issues and underlying factors (axial coding) in the practice visit reports as well as enumerate the frequency with which each theme and subtheme was identified.

The University of Calgary Conjoint Health Research Ethics Review Board approved the project.

## Results

Narrative visit reports were examined for 51 family physicians and general practitioners. In Table 1 the group of selected physicians is compared with a longitudinal study of 250 non-selected family physicians and general practitioners who had completed PAR on two occasions [15]. The median time since medical graduation in both cohorts was similar at 28 years (range 7 to 58 years for this study). In comparison with the longitudinal study [15] the group of flagged physicians showed increased proportions of males, international medical graduates, non-certification in family medicine, and rural practitioners. These trends were similar to the findings in previous studies of visits to family practitioners in Ontario [8,16].

**Table 1:** **Demographics of visited PAR physicians, and prior study**[15]**of unselected family physicians and general practitioners (IMG – international medical graduate, CCFP – certificant of the College of Family Physicians of Canada)**

**Percentages**	**Males**	**Canadian**	**IMG**	**CCFP**	**Metro**	**Urban**	**Rural**
Prior study n=250	67	73	27	50	65	11	24
This study n=51	74	46	54	24	48	16	36

Domains of practice performance recognised by factor analysis in the PAR questionnaires [2] are listed in Table 2. In this cohort, as in previous studies of PAR [1,3,5], patient ratings were generally high, all above 4.0; co-worker ratings were in the range 2.9 to 4.9; and medical colleague ratings were in the range 2.7 to 4.6, which provided the lowest mean and also the largest contribution to “flagging” of physicians.

**Table 2:** **PAR questionnaire survey attribute categories**[2]**for 51 family physicians, with summary ratings for medical colleagues, co-workers and patients**

**Colleagues**	**n**	**min**	**max**	**mean**	**std dev**
Clinical competency	51	2.70	4.50	3.69	0.304
Patient interaction	40	3.10	4.60	3.82	0.308
Professional self-management	38	2.80	4.30	3.71	0.288
Consultation communication	29	3.20	4.40	3.80	0.278
**Co-workers**	**n**	**min**	**max**	**mean**	**std dev**
Patient interaction	51	3.10	4.90	4.07	0.405
Co-worker communication	44	2.90	4.80	4.01	0.410
**Patients**	**N**	**Min**	**Max**	**Mean**	**std.dev**
Patient interaction	50	4.00	4.90	4.55	0.219
Information for patients	46	3.70	4.90	4.37	0.246
Personal communication	50	3.60	4.80	4.41	0.271

Several recurring problems affecting the quality of patient care were identified in discussions of practice visit reports by the Assessment Committee. These included consequences of isolation in both rural and urban practice, superficial or cursory consultations resulting in diagnostic conclusions being reached with incomplete clinical evidence, and poor compliance with standards of practice and accepted guidelines. Family practice includes diverse models of care and variable types of patient-physician relationships. In some “walk-in clinic” practices patterns of single complaint episodic care were observed without sufficient attention to background illness or continuity of care. This problem was reported by the ASC to the regulatory limb of the CPSA and this prompted a new enforceable standard of practice for episodic practice.

**Figure 2:**
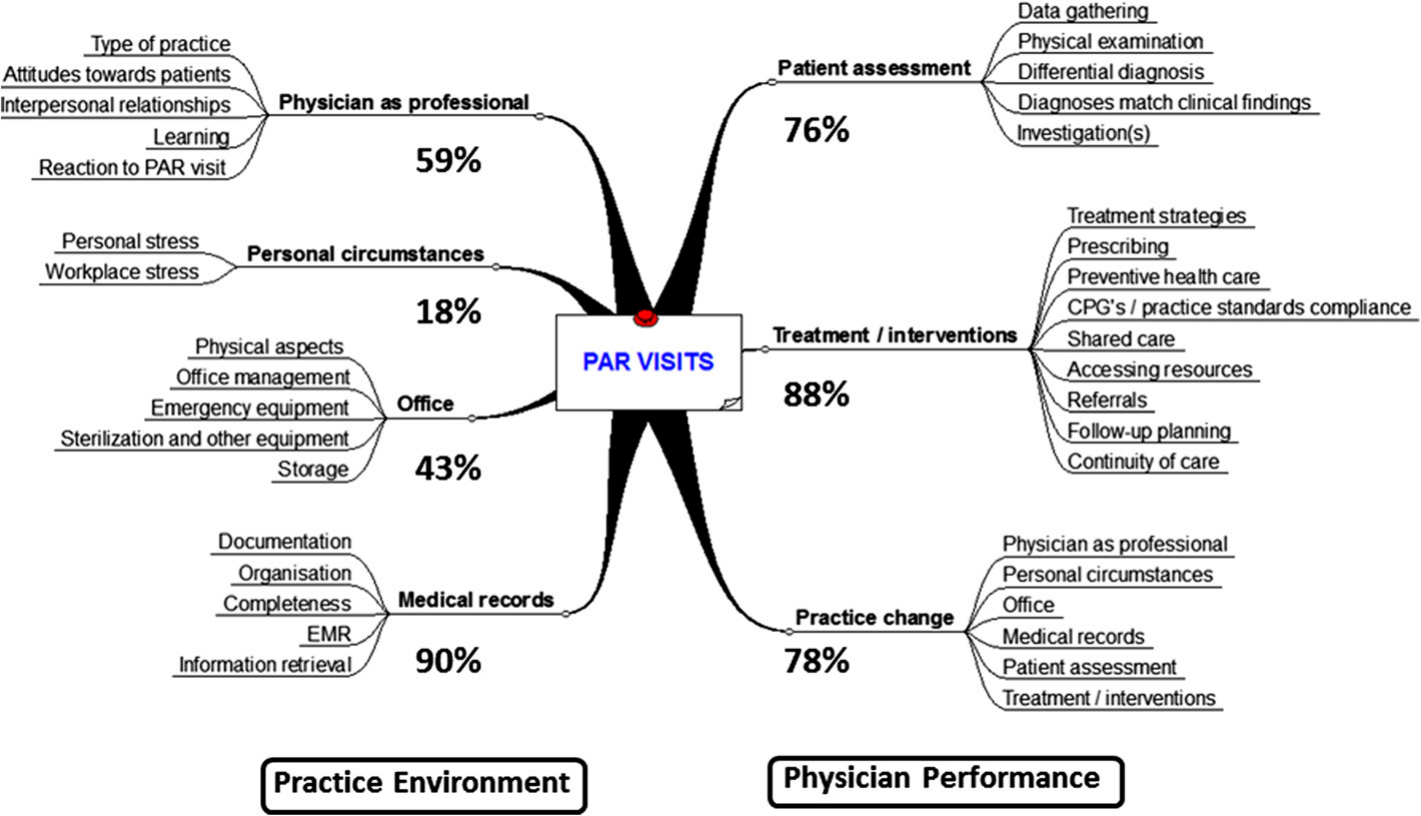
**Themes identified by qualitative analyses of 51 PAR practice visit reports.** Percentages of items requiring practice change or improvement, and categories in which practice change occurred are shown.

Thematic analysis of the visit reports generated two main categories in which concerns were observed – the practice environment and context of the practice, and physician performance and quality of care. Themes and sub-themes within these categories are illustrated in Figure 2, and frequencies of occurrence are shown in Table 3. Information in the categories “physician as professional” and “personal circumstances” was derived from the pre-visit questionnaire and pre-visit conversations between the DPI and the visited physician. The extracts and illustrative examples from the report narratives that follow below are classified according to the themes listed in Table 3, with mention of recommended actions and practice changes that were reported in 40 (78%) of the reports. Minor deficiencies, usually in record keeping, and not requiring further involvement of the PAR program were observed in the other 11 practices.

**Table 3:** **Frequencies of themes and subcategories identified in 51 PAR visit reports and numbers of physicians who made practice changes**

**A. Practice environment**		**B. Physician performance**	
**1. Physician as Professional n=30 (59%)**	N	**5. Patient Assessment n=39 (76%)**	N
o Type of practice	12	o Data gathering	26
o Attitude towards patients	2	o Physical examination	16
o Interpersonal relationships	22	o Diagnosis and differential diagnosis	26
o Learning	9	o Investigation	21
**2. Personal Circumstances n=9 (18%)**		**6. Treatment/Interventions n=45 (88%)**	
o Personal stress	9	o Treatment strategies/prescribing	36
o Workplace stress	17	o Preventative health care	17
o Reaction to PAR visit	18	o CPGs/practice standards compliance	29
		o Shared care	5
**3. Office n=22 (43%)**		o Accessing resources	13
o Emergency equipment	10	o Referrals	13
o Physical aspects	3	o Follow-up planning	22
o Examination rooms	5	o Continuity of care	16
o Office management	14		
o Sterilization and other equipment	3		
o Storage	10	**7. Practice Changes n= 40 (78%)**	N
		o Physician as Professional	15
**4. Medical Records n=46 (90%)**		o Personal Circumstances	5
o Completeness	37	o Office	12
o Documentation	12	o Medical Records	31
o Organization	12	o Patient Assessment	21
o EMR	19	o Treatment/Interventions	31
o Information retrieval	8		

### A. Practice environment and context

#### Physician as professional

*Type or pattern of medical practice* was found to influence the ability of physicians to provide continuity of care. In “walk-in clinic” practices some physicians were not informing patients that their professional role was confined to that single episode of care. Deficiencies were seen in identifying the physician who would continue to provide care or deal with the results of investigations or assume responsibility for chronic illnesses (e.g., hypertension, diabetes). *Recommended actions and practice changes* included making sure that patients were notified of boundaries between the provision of episodic care and the need for preventative and continuing care in chronic illness.

*Attitudes to patients* were found unsatisfactory in strategies for patient-centred care, including managing patients with abrasive or demanding personalities.

*Recommended actions and practice changes* included use of techniques and models for behavioral change. In providing patient-centered care it was emphasised that ways should be found to increase feedback from patients about their expectations and concerns and to generate an agenda in talking with patients. The principles of active listening were outlined: agree, acknowledge and assist, and asking appropriate questions such as how can I help?

*Interpersonal relationships* were observed to be negatively affected in practice circumstances that isolated physicians from colleagues either because of differing styles of practice or geographic isolation. Isolation was of particular concern for physicians who were new to Canada, especially in rural or geographically remote practices where opportunities to interact socially and professionally with colleagues are often limited. The PAR category of communication with colleagues and co-workers was one of the marker flags for physicians found to have inter-personal relationship difficulties. *Recommended actions and practice changes* to improve communication with patients, colleagues and co-workers included enrollment in patient-physician communication courses and use of electronic guidance materials. Participation in out-of-hours on-call schedules and joining medical discussion groups were recommended for isolated physicians.

*Learning and knowledge deficiencies* were sometimes associated with practice environments that had insufficient access to electronic sources of information and educational materials. *Recommended actions and practice changes* included regular use of internet sources of medical information and becoming more skilled in how to use point-of-care resources to answer questions that arise in routine practice.

#### Personal circumstances

*Personal stress and workplace stress* were encountered in physicians working excessively long hours in their practice and in on-call responsibilities. In some instances personal and social stresses in marriage or in financial matters adversely affected performance. The PAR survey self-assessment questionnaires include an item on personal management of stress, and responses to this were found to be a marker for difficulties in personal circumstances. Physical disability including visual impairment and physical incapacity required expert advice and support.

*Recommended actions and practice changes* included avoidance of stressful patterns of practice, strategies to reduce practice isolation, improved time and work management, and regular vacations. Some physicians were advised to reduce the scope of their practice responsibilities or to contact the personal and family support program offered by the provincial medical association.

*Reactions to the PAR program* included resentment of implied criticism and resistance to the suggestion of practice visits. It was not unusual that physicians felt threatened by the process, for which a useful remedy was detailed explanation that the program is educational and not disciplinary. Fears were usually assuaged by reassurance regarding the confidential supportive nature of the program, and its separation from the disciplinary functions of the CPSA.

#### Office

*Physical aspects.* Documents for practice visits [2] require preliminary inspection of the practice premises. The majority of family physicians in Alberta are self-employed. It is their responsibility to ensure that equipment and medications for emergencies are readily available in their practice. *Recommended actions and practice changes* included appropriate precautions for dealing with needles and sharp implements, and to appoint a member of the office staff to be responsible for safety.

*Office management* problems included communication problems with inconsistent handling of correspondence and procedures for dealing with abnormal investigation results, returning patient telephone calls, scheduling of appointments including provision for urgent consultations. Maintenance of privacy and confidentiality within the office was poor in some practices, including security of records, prescriptions, outgoing faxes, and general compliance with confidentiality legislation. Other issues in office facilities included physical access for disabled patients, inefficient patient flow within the office, effectiveness of sterilization procedures, and retaining stored medications in the office beyond the expiration date. *Recommended actions and practice changes* included working with colleagues to produce a system to deal with incoming correspondence and laboratory results, making time in the day to regularly deal with telephone calls, arranging for clinical cross-coverage with colleagues, and increasing the number and duties of support staff.

#### Medical records

*Documentation problems* included poor legibility of handwritten notes, excessive use of abbreviations, and absence of patient health context summaries (problem lists). In some practices with sharing of patients amongst physicians, visit notes were inadequate. Specific omissions included social and occupational history, failing to initial laboratory results prior to filing, and absence of consent prior to office procedures. Disorderly organisation of paper records was more frequent than in electronic medical records (EMR), but some physicians had inefficient use of their installed EMR because of adoption and implementation problems. *Recommended actions and practice changes* for documentation in paper records included improved organisation for efficient data retrieval, use of problem lists and detailed summaries, color coding of important elements such as medication lists, identification of the physician making an entry, tagging charts with abnormal findings or results, use of flow sheets and care maps for chronic conditions such as diabetes, hypertension and anticoagulation therapy, and careful documentation of all prescribed medications with dosages. Physicians were instructed to always include sufficient information in the record to enable another clinician to understand the assessment and rationale for management choices, and to be consistent in recording the history, examination, working diagnosis and follow-up plan. *Recommended practice changes* for EMR included increased usage to deal with handwriting legibility, use of electronic templates and graphics, gathering all information in one place in the electronic record, and use of EMR as an adjunct for clinical decision-making. Technical issues raised included secure login, regular backup of the electronic system, and vigilance for dictation errors. To confirm changes in paper and electronic record-keeping visited physicians were asked to fax copies of their revised records to the DPI, with patient identifiers obscured or removed.

### B) Physician performance and quality of care

#### Patient assessment

*Data gathering* was deemed unsatisfactory by physicians who failed to inquire beyond presenting symptoms without looking for underlying causes. In patients requiring continuity of care there were failures to record risk factors for common diseases or omission of social history and occupational history. *Recommended actions and practice changes* included development of short questionnaires for patients to complete in the waiting room, streaming of patients according to risk categories and having staff record relevant information from the history before the patient is seen, regular recall for periodic health examinations, addition of summary sheets with important personal data, increased detail in documentation of potentially serious problems such as chest pain and headache, appropriate use of the DSM IV diagnostic classification, and allocation of extra time to assess complex patients.

*Physical examination* data deficiencies included key physical findings that had not been found by the physician or apparently had been found but not documented, with the result that the basis for diagnosis was unclear. In records showing diagnoses that did not match clinical findings there was often premature closure of diagnostic process and prescription of medications such as antibiotics with insufficient justification. In practices using shared records there was failure to document the names of other physicians who had been involved with the patient. Unsatisfactory examples from patient examination records were absence of periodic blood pressure measurements in treated hypertensive patients, failure to use cognitive ability tools in older patients, and not using growth charts in pediatric patients. Other concerns included failure to perform sensitive examinations (with chaperone when appropriate), and failure to document patients’ refusals for investigation or treatment after provision of advice.

*Investigation* deficiencie*s* included routine ordering of batteries of multiple tests, and excessive ordering of investigations that are not evidence-based for periodic health examinations. In patients with chronic illnesses such as diabetes and hypertension, protocols were not followed for regular laboratory monitoring, such as hemoglobin A1C and renal function. *Recommended actions and practice changes* included routine record audit to assess the appropriateness of laboratory investigations as an indicator of quality of medical care provided in the practice.

#### Treatment and interventions

*Treatment strategies and prescribing* deficiencies included insufficient details when matching treatments to diagnoses, absence of therapeutic plans, concerns about usage of narcotics, benzodiazepines and antibiotics, and poor management of non-malignant pain. The use of prescriptions to abbreviate patient visits carried risks of missing potentially important clinical findings. *Recommended actions and practice changes* included advice to continue treatment and supervision for patients awaiting specialist consultations. Physicians were advised to ensure that active medication lists correspond with problems on the medical record and the reasons for management strategies that were adopted; to always record drug names, quantities and dosages and number of refills when writing prescriptions; and to provide evidence of discussion with patients about the risks and benefits of potent medications, especially for long-term treatment. They were advised to be knowledgeable in clinical pharmacology including benefits, adverse effects and interactions of drugs, to follow clinical practice guidelines for common infections and antibiotic usage, and to exercise caution in prescribing for patients with addictive substance problems.

*Preventive health care* problems included inconsistent implementation of procedures such as Pap testing, influenza and pneumococcal vaccination, and screening for colorectal and breast cancer. Preventive approaches for chronic illness were deficient or missed for common conditions such as diabetes, hypertension, dyslipidemia, asthma and depression. *Recommended actions and practice changes* drew attention to national or provincial clinical practice guidelines for common problems in family practice, identification of risk factors and risk scores, for example in cardiac health, lifestyle management and healthy behaviors.

*Shared care*. Despite most family physicians working in groups, and availability of government-funded support for additional professional staff, collaborative patterns of care with physicians and other staff were often uncoordinated and not patient-centred. *Recommended actions and practice changes* included to always make the patient the central figure in the work of the practice. Physicians were advised to provide health promotion, illness prevention, monitoring and screening in conjunction with the health care team. Visits dedicated to the management of aspects of chronic diseases were advised, including other members of the team. It was noted that each encounter must have sufficient information recorded to allow other physicians and team members to understand assessment and management strategies.

*Accessing resources - recommended actions and practice changes* included appropriate distribution of information about health services available within the local community, working with allied health professionals, volunteer organisations and societies, and access to the pharmacy information network.

*Referrals*: Concerns were noted when physicians failed to recognise that a patient’s problem was beyond their usual scope of expertise, and the related situation of physicians not continuing to provide care after patients had been referred to specialists. Information provided in referrals to specialists was often incomplete, for example in providing details of preceding treatment. *Recommended actions and practice changes* emphasised the need to improve the information provided with requests for specialist consultations, and to retain copies of referral letters. Continuing care of the patient is required while waiting to be seen by the consultant, and appropriate follow-up with advice after the consultation was necessary. Increasing the scope of commonly required services provided by the physician was suggested to decrease the number of external consultations.

*Follow-up:* There was particular concern in episodic and walk-in clinic practices regarding failures to plan for further care of patients. This was seen as a serious safety issue in monitoring chronic illnesses and renewal of medications. *Recommended actions and practice changes* included instructions for patients to be recorded at the end of each visit report and to provide adequate written documentation to support safe follow-up of patient care whether or not the patient returns to the clinic and the same physician is the provider of subsequent care.

*Continuity of care* was found to be a problem in physician-patient relationships in all types of practice, and was identified to some extent in various categories during PAR practice visits. *Recommended actions and practice changes* included clinical, laboratory and preventive strategies and target setting for chronic illnesses, standardised flow sheets, vigilant monitoring of prescription renewals for patients on long-term medications, and ensuring continuity of care for patients seen in on-call and episodic circumstances.

## Discussion

This study examined 51 detailed reports for family physicians and general practitioners who were flagged during the PAR MSF process and then had a practice visit. Potential risk factors for performance difficulties were evident in the demographic differences between the group of flagged physicians and the most recent PAR longitudinal study [15], including non-certification in family practice and medical graduation outside Canada. The important influence of organisational or systemic risk factors [16] was also apparent, for example where there was isolation with limited access to collegial networks, and in the walk-in clinic environment that was observed to sometimes provide insufficient or superficial care.

While MSF has been described as more helpful in the CanMEDS [17] professional roles of communicator, collaborator, and professional, this study demonstrates that MSF also identifies physicians for whom the role of medical expert may be of concern. The practice visit reports identified problems in medical records, patient assessment and treatment/interventions for 90%, 76% and 86% of physicians respectively.

During initial development of the PAR program 83% of 255 volunteer physicians reported that their MSF feedback data stimulated contemplation of change, and 66% reported initiation of change in at least one aspect of their practice [4]. In the implemented program post-PAR survey questionnaires [18] are distributed 3 months after physicians receive their PAR report. In 2011, including the period of the present study, 42% of physicians responded to the post-PAR survey, of whom 70% felt that PAR feedback was valuable, and 40% to 50% (in different types of practice) reported that they had made changes in at least one aspect of practice, most often in aspects of direct patient care and communication. The study of flagged family physicians and general practitioners found that areas for improvement were proposed in some aspect of practice for almost all visited physicians, and change was made and confirmed for 78% of physicians. The final steps of working with individual physicians to facilitate or reinforce change through discussion and subsequent confirmation of practice changes have not been previously reported for the PAR program.

This retrospective qualitative analysis of PAR practice visit reports has provided evidence that MSF questionnaires identify physicians whose practices can benefit from additional assisted review. These findings substantiate the intended principles of the PAR program [1], and also confirm the proposition [19] that routine assessment of the quality of care in family practice can identify strengths and weaknesses, prompt changes in the quality and efficiency of care, and contribute to establishment of standards of care.

There are a number of contextual limitations in the present study. In Canada the assessment of performance by medical regulatory authorities has mostly focused on physicians considered to be at risk for performance difficulties or for whom there has been evidence of poor performance requiring competence remediation [20-22]. In developing the PAR program the CPSA chose an innovative path, requiring participation of all clinical physicians, and administratively separated from the complaints and disciplinary functions of the College. The program is funded by annual medical licence dues, and in allocating finite resources, emphasis has been placed on developing robust MSF tools, adapted to 7 varieties of medical practice [2] and a formative educational model of professional development.

A procedural limitation in the present study is that PAR practice visit protocols are not directly linked to MSF questionnaire reporting domains [2]. However, the experience reported in this study has been that PAR flagging provides a credible signal for exploratory dialogue, followed by practice visits for a minority of physicians*.* The program’s sequence of contacts for flagged physicians is designed to progressively focus resources where this may be most opportune to encourage practice reflection and change. It is acknowledged that practice visits for a larger proportion of the flagged physicians, and also non-flagged physicians, would be useful and informative if sufficient resources were available.

## Conclusion

The PAR MSF questionnaires were initially compiled from the expectations of groups of physicians in various areas of practice, as well as the opinions of focus groups of patients and co-workers [1]. Analysis of practice visits provides a *post hoc* perspective and the areas of concern found in this study can be used as an internal quality improvement tool in further modifications to the PAR program.

PAR provides educational feedback for practice reflection and quality improvement and in this manner may reduce risks associated with poor performance. It is recognised that the ultimate value of this approach depends on demonstration of both beneficial improvement in medical care as well as the quantitative validity of the instruments [23].

The increasing adoption of MSF methodology in medical regulatory and service environments is likely to better define its roles in differing circumstances and in conjunction with other performance assessment tools [24].

The extent to which practice changes occur following provision of MSF will be influenced by the acceptability of information perceived to be negative [25] and the manner in which positive motivation is encouraged [26]. The current study provides descriptive evidence for the value of supplementary assessment, conversation and individualised guidance for flagged physicians in the PAR program.

## Competing interests

The authors declare that they have no competing interests.

## Authors’ contributions

All authors contributed to the design of the study and the identification of performance themes. NF is the Director of Practice Improvement (DPI) in the PAR program and was responsible for organisation of practice visits and communication with visited physicians. RL led the writing process and all authors commented on drafts and approved the final manuscript.

## Pre-publication history

The pre-publication history for this paper can be accessed here:

http://www.biomedcentral.com/1472-6920/13/121/prepub
